# Report of the Select Committee to Whom Was Referred the Subject of the Petition of William T. G. Morton of Boston, Asking Remuneration for the Discovery of the Anaesthetic Influence of Sulphuric Ether

**Published:** 1849

**Authors:** 


### ARTICLE III.

*Report of the Select Committee to whom was referred the subject of the petition of William T. G. Morton of Boston, asking remuneration for the discovery of thc Anaesthetic influence of Sulphuric Ether.* Made to the H. R. United States, by Dr. EDWARDS, Chairman.

This report argues at considerable length the claims of Drs. Jackson and Morton, to the discovery of an agent capable of preventing pain during surgical operations, but does not notice Dr. Horace Well's previous discovery and announcement of the fact in Boston, that Nitrous Oxide will have that influence, perhaps because the friends of the other persons and not those of Dr. Wells were pressing their claims to the discovery.

We think the disputation about the question of priority, is likely to exert an influence upon the reputation of the claimants, similar to that of taking out a patent for it.

The conclusions of the report are more favorable to the claims of Dr. Morton as he put to the test of experiment the agent. E.

